# Assessment of the Knowledge and Attitudes of Saudi Mothers towards Newborn Screening

**DOI:** 10.1155/2015/718674

**Published:** 2015-10-12

**Authors:** Ayman Al-Sulaiman, Altaf A. Kondkar, Mohammad Y. Saeedi, Amal Saadallah, Ali Al-Odaib, Khaled K. Abu-Amero

**Affiliations:** ^1^Department of Genetics, King Faisal Specialist Hospital and Research Center, Riyadh 12713, Saudi Arabia; ^2^Department of Ophthalmology, College of Medicine, King Saud University, Riyadh 11411, Saudi Arabia; ^3^Glaucoma Research Chair, Department of Ophthalmology, College of Medicine, King Saud University, Riyadh 11411, Saudi Arabia; ^4^Ministry of Health, Hereditary and Chronic Diseases Control Department, Riyadh 11176, Saudi Arabia

## Abstract

*Objective*. To assess the attitude and knowledge of the Saudi mothers toward newborn screening (NBS) program. *Methods*. A total of 425 Saudi women (only mothers who have at least one pregnancy) participated in the study from different regions in Saudi Arabia and completed the structured questionnaire which sought their views on the NBS services. *Results*. A majority of the participating women (91.1%) supported the NBS program and felt it was very important and useful. However, knowledge of NBS was found to be very limited and only 34.6% knew that NBS was a test to detect genetic disorders. A lack of communication and counseling to NBS clients by health authorities offering screening is implied. *Conclusion*. In general, there is a positive attitude towards the NBS program among Saudi women. However, they have several concerns to improve the availability of medication and formulas, genetic counseling, medical interventions, communication, education materials, and awareness.

## 1. Introduction

With advances in DNA technologies genetic testing has rapidly evolved from bench to bedside in diagnosis of inherited disorders, carrier testing, prenatal diagnosis, and newborn screening (NBS). NBS represents one of the major advances of the past century in child health that aimed at early identification and management of presymptomatic congenital disorders in affected newborns thereby reducing infant morbidity and mortality [1]. NBS national services have expanded over the years in both the developed and developing nations and efforts are being made to make this into a major public health program [2, 3]. Accordingly, this important preventative program has been implemented by the Ministry of Health (MOH) in Saudi Arabia (since July 2005) and offers screening of infants for 16 known and preventable biochemical and endocrine genetic disorders [4].

Saudi Arabia is an Islamic nation comprising five large provinces. The Western Province which includes the holy cities of Makkah and Medina, the Central Province which includes the Saudi capital Riyadh, the Eastern Province, and the Northern and Southern Provinces. The successful implementation of nationwide preventive genetic program can be affected by various factors including cultural, traditional, and religious issues [5–7]. It is therefore critical to realize that NBS if intended as a national service needs to secure several areas from which it could spring forward. Primarily, the success of any screening program requires public participation and awareness, or else it would remain stagnant or proceed in a staggered, incomplete, irregular mode void of the true benefits intended for a nation [2]. Earlier studies have strongly emphasized the importance of educating parents and health care professionals about NBS and its practices [8, 9].

NBS has been implemented in a number of different disease contexts worldwide [2, 10, 11]. The evaluation studies of this service have mainly focused on the appraisal of treatment outcomes. The social and psychological impacts of the screening program as well as attitudes towards it have been the focus of only a few studies [12–17], with no studies done in Saudi Arabia so far. Since the success of NBS programs depends largely on parental participation and approval, our study assessed the attitudes and knowledge of the Saudi women towards the NBS program and their psychological impact in an attempt to evaluate the outcome of the NBS program offered by the MOH in Saudi Arabia.

## 2. Methodology

### 2.1. The Study Population

We conducted a cross-sectional survey of a total of 425 women from different regions in Saudi Arabia. All the women participating in the study have at least one pregnancy. Of the 425 participants, 175 were mothers from the clinics of the Clinical Genetics Department at King Faisal Specialist Hospital and Research Center (KFSH&RC) with at least one affected child screened by the NBS program. Additionally, 250 participants were pregnant women from the Obstetrics and Gynecology clinic at KFSH&RC. These participants completed a structured questionnaire which sought their views on the offered NBS services. The study was approved by both the research advisory and ethical committee of KFSH&RC, Riyadh, Saudi Arabia. All information obtained from this study was treated with confidentiality.

### 2.2. Questionnaire and Analysis

To fulfill the research objectives and answer the research questions appropriately, different questionnaires were designed to identify the subject's knowledge, attitudes, and concerns regarding the NBS programs. Structured questionnaires were selected as the most appropriate instrument because of their capacity to generate quantifiable data from all the studied groups. These approaches made it possible to measure what a person thought (attitudes and beliefs), what a person knew (knowledge or information), and what a person liked and disliked about the NBS service (satisfaction and concerns). The questionnaire was sent to the corresponding coordinators of the NBS program at the various participating hospitals offering NBS services around the Kingdom of Saudi Arabia via email. These coordinators would in turn provide the questionnaire to the participants, collect it back, and return it to KFSH&RC in Riyadh. The questionnaires were coded and entered into the Statistical Package for the Social Sciences (SPSS) program. All data were analyzed and summarized in the form of frequencies and percentages.

## 3. Results

Of the 425 participating women, 326 (76.7%) had their delivery at the hospital and in almost half, 201 (47.3%) women, their child was tested through the NBS program.

### 3.1. Demographics

The participants were found to be young in age (mean age = 30.8 years) and well educated, and 60.5% were housewives. The demographic details of the participating mothers from different regions in Saudi Arabia are tabulated in Table 1.

### 3.2. Attitude toward NBS

On a very positive note, 387 (91.1%) of the participating women supported the program and felt that NBS was very important and useful to the parents.

### 3.3. Knowledge of the Participants about NBS

Participants were asked if they knew what NBS testing was all about before or after the delivery, for example, “*Have you had any previous knowledge about newborn screening?”*

Only 96 (22.6%) women replied affirmatively while a majority of 300 (70.6%) women replied negatively, whereas 29 (6.8%) women reported they were not sure. Furthermore, only 147 (34.6%) women indicated that the test was done for some genetic disorder, whereas 266 (62.6%) women indicated NBS as a blood sample test; and a huge proportion, 360 (84.7%) women, believed that NBS testing requires samples from both the parents.

### 3.4. Treatment Satisfaction

Of the 175 responders whose babies were tested by the NBS program, 100 (57%) women reported that NBS treatment was offered at the right time but overall only less than half, 84 (48%) women, were satisfied with the medication and treatment offered by the hospitals.

### 3.5. Service Satisfaction

Participant's satisfaction of receiving the genetic testing results from the hospital was also evaluated, for example,* “You received the results of newborn screening with satisfaction?”*

Overall, of the 201 babies of the participating mothers that went through the NBS testing, 115 (57.2%) reported to be satisfied. It was found that the mode of communicating the results varied and was done by different medical staff. Of the 146 responders, in 56 (38.3%) cases the results were communicated by a doctor, 29 (19.9%) by a nurse, 11 (7.5%) by coordinators, and 35 (24%) over the telephone, and 15 (10.3%) were not sure.

### 3.6. Requests for Repeat Sampling

This query was answered by 162 women. Of these, it was observed that no repeat sample was requested in 82 (50.9%) cases; repeat sampling was requested twice in 27 (16.8%) cases, thrice in 33 (20.5%) cases, and more than three times in 20 (12.4%) cases.

### 3.7. Refusal to Resample

Of the 153 women that responded to this question it was found that 133 (87%) women accepted as opposed to 32 (20.8%) women that refused to resample requests.

### 3.8. Waiting Period for the Results

The waiting period to receive the results varied from 2 to 5 days in 48 (11.2%) participants, varied from 6 to 10 days in 55 (12%), and was more than 10 days in 322 (75.8%) participants.

### 3.9. Need for Genetic Counseling

To assess the need for genetic counseling the question asked was “*Do you think that it is important to have genetic counseling before and after newborn screening?*”

Of the 425 participating women, 401 (94.4%) women believed genetic counseling to be a very important service before and after the NBS testing.

## 4. Discussion

This study is the first attempt to assess parental experience of NBS testing among Saudi women. The inauguration of the hypothyroidism NBS program in Saudi Arabia 17 years ago to detect and prevent hypothyroidism was a milestone in the management of this group of inherited genetic disorder [18]. It was therefore not surprising to see from our survey that most mothers supported NBS and believed it to be a very important screening program.

The knowledge and understanding of NBS among the participating mothers were found to be low. It was found that only 22.6% had some background about the nature of the test and almost the third of the participants were aware of neither the source of sample required nor the type of diseases to be tested. In fact, 84.7% reported that blood sample is taken from both parents. In addition, 20.8% refused requests to resample. Lack of knowledge can imply lack of information about the nature of the test, the various aspects of genetic testing, including occurrence of false positives and negatives, and the need for repeat testing even to confirm the obtained results. Such issues can lead to problems such as anxiety in mothers receiving requests for repeat testing for their babies unaware that the most likely reason could be inadequate original sample and not a positive test for any disease. An earlier study has shown that false-positive screening results can increase the risk of parental stress and affect parent-child interaction [19]. Obstetrician, pediatrician, and nurses should be involved in the education of parents regarding the availability of NBS testing, the benefits of early detection of disorders for which screening is performed, the risks that exist for newborn infants who do not receive screening, the process of screening, and need for follow-up [20].

Despite the fact that the Saudi mothers supported NBS the study highlighted some of their concerns and areas of improvisation in the NBS program. It was found that only 57% were satisfied about receiving the result from the hospital and that the waiting period for the results was considerably high. The primary focus of the follow-up program should be to locate infants with abnormal screening results and facilitate timely diagnostic testing and management to communicate with the parents. It is a highly crucial time between the staff of the NBS program and the parents if mortality, morbidity, and disabilities are to be avoided. In addition, it was found that only 48% were satisfied with the medications offered by the hospitals. Appropriate and timely dietary and medical interventions are the core to the successful application of the NBS program. The goal should always remain to provide care that is accessible, family centered, continuous, comprehensive, coordinated, compassionate, and culturally competent [21].

In summary, the findings of the study suggest that there is high acceptance of NBS among Saudi women and there is a considerable need to bridge the communication between the medical community and the parents to increase their awareness. It would be important to address parental attitude towards consent for NBS testing and their views on the impact of NBS diagnosis of a genetic disease or carrier status in their infants. However, it is worthwhile to note that mothers with greater knowledge of NBS have been shown to be least likely to provide consent to NBS screening for their babies and increased compliance to follow-up [22]. Although much progress has been made, implementation of NBS as a successful national plan in a way that may eventually lead to a true evidence-based approach will require collaboration, genetic counseling, education, adequate funding for research, and an infrastructure that provides a larger role for a central body.

## Figures and Tables

**Table 1:** Demographic details of the participating women.

Variables	Participants (*n* = 425)
Age range (years)	18–48
Mean age	30.8
Education, number (%)	
Intermediate or below	112 (26.5%)
High school/diploma	151 (35.6%)
University & above	132 (31.1%)
Occupation, number (%)	
Housewife	257 (60.5%)
Education & health	101 (23.6%)
Government sector	17 (4.0%)
Others	49 (11.6%)
Regional Distribution, number (%)	
Northern	49 (11.6%)
Southern	75 (17.7%)
Eastern	71 (16.7%)
Western	46 (10.8%)
Central	180 (42.5%)

## References

[1] Guthrie R. (1992). The origin of newborn screening. *Screening*.

[2] Therrell B. L., Padilla C. D., Loeber J. G. (2015). Current status of newborn screening worldwide: 2015. *Seminars in Perinatology*.

[3] Wilcken B., Wiley V. (2008). Newborn screening. *Pathology*.

[4] Afifi A. M., Abdul-Jabbar M. A. (2007). Saudi newborn screening. A national public health program: needs, costs, and challenges. *Saudi Medical Journal*.

[5] Al Sulaiman A., Suliman A., Al Mishari M., Al Sawadi A., Owaidah T. M. (2008). Knowledge and attitude toward the hemoglobinopathies premarital screening program in Saudi Arabia: population-based survey. *Hemoglobin*.

[6] Alsulaiman A., Hewison J., Abu-Amero K. K., Ahmed S., Green J. M., Hirst J. (2012). Attitudes to prenatal diagnosis and termination of pregnancy for 30 conditions among women in Saudi Arabia and the UK. *Prenatal Diagnosis*.

[7] Alsulaiman A., Abu-Amero K. K. (2013). Parent's attitude toward prenatal diagnosis and termination of pregnancy could be influenced by other factors rather than by the severity of the condition. *Prenatal Diagnosis*.

[8] Kemper A. R., Fant K. E., Clark S. J. (2005). Informing parents about newborn screening. *Public Health Nursing*.

[9] Hargreaves K. M., Stewart R. J., Oliver S. R. (2005). Informed choice and public health screening for children: the case of blood spot screening. *Health Expectations*.

[10] Büyükgebiz A. (2013). Newborn screening for congenital hypothyroidism. *Journal of Clinical Research in Pediatric Endocrinology*.

[11] Peterson C., Grosse S. D., Glidewell J. (2014). A public health economic assessment of hospitals' cost to screen newborns for critical congenital heart disease. *Public Health Reports*.

[12] Christie L., Wotton T., Bennetts B. (2013). Maternal attitudes to newborn screening for fragile X syndrome. *American Journal of Medical Genetics, Part A*.

[13] Faulkner L. A., Feuchtbaum L. B., Graham S., Bolstad J. P., Cunningham G. C. (2006). The newborn screening educational gap: what prenatal care providers do compared with what is expected. *American Journal of Obstetrics and Gynecology*.

[14] Kim S., Lloyd-Puryear M. A., Tonniges T. F. (2003). Examination of the communication practices between state newborn screening programs and the medical home. *Pediatrics*.

[15] Parsons E. P., King J. T., Israel J. A., Bradley D. M. (2007). Mothers' accounts of screening newborn babies in Wales (UK). *Midwifery*.

[16] Quinlivan J. A., Suriadi C. (2006). Attitudes of new mothers towards genetics and newborn screening. *Journal of Psychosomatic Obstetrics and Gynecology*.

[17] Davis T. C., Humiston S. G., Arnold C. L. (2006). Recommendations for effective newborn screening communication: results of focus groups with parents, providers, and experts. *Pediatrics*.

[18] Saadallah A. A., Rashed M. S. (2007). Newborn screening: experiences in the Middle East and North Africa. *Journal of Inherited Metabolic Disease*.

[19] Waisbren S. E., Albers S., Amato S. (2003). Effect of expanded newborn screening for biochemical genetic disorders on child outcomes and parental stress. *The Journal of the American Medical Association*.

[20] Larsson A., Therrell B. L. (2002). Newborn screening: the role of the obstetrician. *Clinical Obstetrics and Gynecology*.

[21] Medical Home Initiatives for Children With Special Needs Project Advisory Committee (2002). The medical home. *Pediatrics*.

[22] Truog R. D. (1996). Is ‘informed right of refusal’ the same as ‘informed consent’?. *Journal of Clinical Ethics*.

